# Ecotoxicological Effects of Emerging Contaminants on Aquatic Species

**DOI:** 10.3390/toxics12080567

**Published:** 2024-08-03

**Authors:** Thiago Lopes Rocha, Luís Manuel Félix, Davi Farias

**Affiliations:** 1 Laboratory of Environmental Biotechnology and Ecotoxicology, Institute of Tropical Pathology and Public Health, Federal University of Goiás, Goiânia 74055-110, Brazil; 2 Centre for the Research and Technology of Agro-Environment and Biological Sciences (CITAB), University of Trás-os-Montes and Alto Douro (UTAD), Quinta de Prados, 5000-801 Vila Real, Portugal; lfelix@utad.pt; 3 Institute for Innovation, Capacity Building and Sustainability of Agri-Food Production (Inov4Agro), UTAD, Quinta de Prados, 5000-801 Vila Real, Portugal; 4 Laboratory for Risk Assessment of Novel Technologies, Department of Molecular Biology, Federal University of Paraiba, João Pessoa 58050-085, Brazil; davi@dbm.ufpb.br

Emerging contaminants (ECs), also known as contaminants of emerging concern (CECs), are defined as synthetic or naturally occurring chemicals or biological agents that are not commonly monitored in the environment but have the potential to enter the environment and cause known or supposed adverse ecological and human health effects. ECs include nanomaterials, micro(nano)plastics, pharmaceutical and personal care products (PPCPs), emerging pathogens and their derivatives, pesticides, and other chemicals that are detected in different environmental compartments (the atmosphere, soils, and aquatic systems), but whose environmental risk is not fully understood [1,2].

Advances in ecotoxicology have led to an ongoing paradigm for the environmental assessment of ECs, particularly in the context of One Health. The One Health approach aims to connect environmental, human, and animal health in an interdisciplinary and collaborative manner. Therefore, the analysis of the ecotoxicological effects of ECs in the 21st century should not be carried out in a fragmented and isolated manner [3].

Given the increasing amount of ECs being released into the environment and concerns about their hazardous effects on human, animal, and environmental health, this Special Issue of *Toxics* is dedicated to the investigation of the ecotoxicological effects of emerging contaminants on aquatic species using in silico, in vitro, and in vivo approaches. This Special Issue contains four original research articles and two review articles.

The article by Teixeira and colleagues assessed the embryotoxic effects of diflubenzuron (DFB) and pyriproxyfen (PPF), alone and in mixtures, in the freshwater fish *Danio rerio*. The results show neurotoxic effects, behavioral changes, and redox imbalance, highlighting the complexity of EC mixtures and indicating potentiating effects on developing zebrafish.

The study by Guabloche and colleagues aimed to assess the concentration of potentially toxic and essential elements in the muscle and liver tissues of the lorna drum, *Sciaena deliciosa*, as well as their presence in the water and sediments of the coastal zone of Callao Bay, Peru. Their results confirm the importance of aquatic organism studies to assess the complex interactions between aquatic pollution, water quality, and animal health and indicate that further studies are needed to explore the long-term effects of pollution on organisms.

Souza and colleagues used network toxicology and molecular docking to identify molecular targets and mechanisms of toxicity in organophosphate pesticides (OPs). From the results, the authors identified the hub nodes HSP90AA1, EGFR, MET, and SRC as potential targets for OPs. Furthermore, the potential neurodevelopmental toxicity induced by OPs was associated with signal transduction, axon guidance, cellular response to stress, and NMDAR activation. Interestingly, the authors confirmed that network toxicology and molecular docking can reveal interactions between chemicals and their predicted targets, helping to determine their ecotoxicological effects.

Rohner and colleagues analyzed the potential accumulation of anti-seizure medication (ASM) and neuropathological changes in brain samples of Eurasian otters (*Lutra lutra*). The presence of several ASMs was confirmed, but no histopathological changes were observed. The authors also showed that the post-mortem examinations of top predators provide valuable insights into the health of populations and ecosystems.

Finally, the Special Issue also includes two review articles on the ecotoxicological effects of ECs on aquatic organisms. Martins and colleagues reviewed the hepatotoxicity of 2,4-dichlorophenoxyacetic acid (2,4-D) and showed that this herbicide decreases the antioxidant capacity and induces changes in energy metabolism, lipids, liver functions, and xenobiotic metabolism. Overall, 2,4-D-induced hepatotoxicity was mainly associated with decreased antioxidant capacity and oxidative stress. In addition, Lawrence and colleagues reviewed the scientific literature and showed that the sensibility of zooplankton organisms to microplastics is evident from laboratory studies, but the mechanism of action, toxicity, and field studies are scarce.

This collection of articles presents the current knowledge and trends in the ecotoxicology of ECs. We hope that this Special Issue will contribute to the advancement of our knowledge on the ecotoxicological effects of ECs on aquatic organisms and their impact on environmental, animal, and human health.
